# Case report: Episodic ataxia without ataxia?

**DOI:** 10.3389/fneur.2023.1224241

**Published:** 2023-10-26

**Authors:** Andrea Gaudio, Fabio Gotta, Clarissa Ponti, Francesca Sanguineri, Lucia Trevisan, Alessandro Geroldi, Serena Patrone, Chiara Gemelli, Corrado Cabona, Guja Astrea, Chiara Fiorillo, Stefano Gustincich, Marina Grandis, Paola Mandich

**Affiliations:** ^1^IRCCS Ospedale Policlinico San Martino—UOC Genetica Medica, Genova, Italy; ^2^Department of Neuroscience, Rehabilitation, Ophthalmology, Genetics and Maternal and Child Health, University of Genova, Genova, Italy; ^3^IRCCS Ospedale Policlinico San Martino—SS Centro Tumori Ereditari, Genova, Italy; ^4^IRCCS-Ospedale Policlinico San Martino—UOC Clinica Neurologica, Genova, Italy; ^5^IRCCS-Ospedale Policlinico San Martino—UOC Neurofisiopatologia, Genova, Italy; ^6^IRCCS Fondazione Stella Maris, Pisa, Italy; ^7^IRCCS Istituto Giannina Gaslini—UOC Neuropsichiatria Infantile, Genova, Italy; ^8^Department of Neuroscience and Brain Technologies, Istituto Italiano di Tecnologia, Genova, Italy

**Keywords:** hereditary myopathies, UBR4, HSPG2, episodic ataxia, genetic modifiers, genetic testing

## Abstract

Hereditary myopathies represent a clinically and genetically heterogeneous group of neuromuscular disorders, characterized by highly variable clinical presentations and frequently overlapping phenotypes with other neuromuscular disorders, likely influenced by genetic and environmental modifiers. Genetic testing is often challenging due to ambiguous clinical diagnosis. Here, we present the case of a family with clinical and Electromyography (EMG) features resembling a myotonia-like disorder in which Whole Exome Sequencing (WES) analysis revealed the co-segregation of two rare missense variants in *UBR4* and *HSPG2*, genes previously associated with episodic ataxia 8 (EA8). A review of the literature highlighted a striking overlap between the clinical and the molecular features of our family and the previously described episodic ataxias (EAs), which raises concerns about the genotype–phenotype correlation, clinical variability, and the confounding overlap in these groups of disorders. This emphasizes the importance of thoroughly framing the patient's phenotype. The more clear-cut the diagnosis, the easier the identification of a genetic determinant, and the better the prognosis and the treatment of patients.

## 1. Introduction

Episodic ataxias (1) and hereditary myopathies (2) are two groups of clinically and genetically heterogeneous neuromuscular diseases. The main molecular determinants are damaging variants in genes encoding for ion channels subunits. Both are characterized by wide phenotypic variability, even intra-familiar, suggesting a possible influence of genetic and environmental modifiers on clinical manifestations.

Hereditary myopathies are a group of muscle disorders characterized by muscle stiffness, cramps, and weakness due to abnormal muscle structure and function. The clinical onset is usually in infancy and can vary among individuals, even within families. Diagnosis involves a combination of clinical evaluation, electromyography (EMG), muscle biopsy, and genetic testing.

EAs are a group of autosomal dominant inherited neuromuscular disorders characterized by variable spells of imbalance and ataxia often accompanied by additional ictal and interictal symptoms (1, 3, 4). The incidence is likely to be less than 1/100,000, but it may be underestimated due to clinical variability and to the incomplete knowledge of genetic causes (4). To date, nine subtypes have been described (EA 1-9) according to shared clinical and genetic features. These characteristics are quite heterogeneous and, in some cases, not fully elucidated (5). The two most common and best characterized forms are EA1 and EA2. The others are rarer and less molecularly depicted, and sometimes described only in single large families and never replicated (EA5) (5–7) (Table 1).

**Table 1:** Overview of the clinical and genetic features of EAs.

**Type**	**Gene**	**Protein**	**AAO**	**Episodes duration**	**Triggers**	**Treatment**	**Ictal symptoms**	**Interictal symptoms**
**EA1** *#160120*	*KCNA1*	Potassium channel K_v_1.1	Early childhood	Seconds-minutes *(hours-days)*	Physical-emotional stress/startle response/abrupt movement	*CBZ (AAZ)*	Vertigo, dysarthria, weakness, tremor, seizure	Myokymia
**EA2** *#108500*	*CACNA1A*	P/Q type Ca^2+^ channel α1 subunit (Ca_v_2.1)	Infancy/Early childhood/*up to 6th decade*	Hours-days	Physical-emotional stress/temperature changes/alcohol—caffeine—chocolate	*AAZ*	Vertigo, dysarthria, weakness, seizure, dystonia, cognitive impairment	Ataxia, nystagmus
**EA3** *#605554*	*1q42*	*Unknown*	Adulthood	1min to 6h	NA	*AAZ*	Vertigo, weakness, visual blurring	Myokymia
**EA4** *#606552*	*Unknown*	*Unknown*	Adulthood	Hours	NA	Unknown	Vertigo	Nystagmus
**EA5** *#613855*	*CACNB4*	P/Q type Ca^2+^ channel β4 subunit	Adulthood	Hours-Weeks	Lack of sleep/fatigue/infections/stress/alcohol	*AAZ*	Vertigo	Ataxia, nystagmus
**EA6** *#612656*	*SLC1A3*	Excitatory amino acid transporter 1	Infancy/Early childhood	Hours-Days	Stress/fever	*AAZ*	Vertigo, weakness, seizure	Ataxia, nystagmus
**EA7** *#611907*	*19q13*	*Unknown*	1st-2nd decade	Hours-Days	Exercise/emotional stress	Unknown	Vertigo, dysarthria, weakness	NA
**EA8** #616055	*UBR4*	E3-Ubiquitin Ligase	Early infancy	Minutes-24h	Exercise/stress/temperature change	CZP	Vertigo, weakness, tremor	Ataxia, nystagmus, myokymia
**EA9** #618924	*SCN2A*	Sodium voltage-gated channel subunit α2	Childhood	Minutes-Days	NA	*AAZ*	Vertigo, weakness	Ataxia

In the column “Type”, #number stands for the MIM associated number.

Italic characters stand for single or few observations.

AAO, age at onset; AAZ, acetazolamide; CZB, carbamazepine; CZP, clonazepam.

Here, we report the case of a family who was referred to our neuromuscular clinic for frequent cramps, especially at night, easy fatigability, muscle weakness, muscle hypertrophy, and twitching predominantly in ocular muscles.

Molecular analysis revealed the presence of two heterozygous missense variants in *UBR4* and *HSPG2*, genes previously reported to co-segregate in a large EA8-diagnosed family (5). The co-segregation of these two variants in our family with a prevalent clinical myopathic phenotype raised concerns about the genotype–phenotype correlation of EA8. This prompted a careful literature review, which highlighted its wide and understudied phenotypic spectrum (Table 2), and the uncertainty in the definition of a precise genetic cause, which suggested a possible, or at least a confounding, overlap between EA8 and other neuromuscular disorders (8).

**Table 2:** Major differences between episodic ataxia 8 described cases and the index cases of the current paper.

**EA8 cases**	**AAO (Y)**	**Episodes duration**	**Episodes frequency**	**Muscle weakness**	**Poor balance**	**Eyes twitching**	**Ataxia**	**Nystagmus**	**Myokymia**	**Tremor**	**EA during pregnancy**	**Response to treatment**
**Conroy et al**.												
I-2	<2	Min-Hours	0/month−3/week	+	+	–	+	+	–	+	NA	CZP
II-6	<2	10 m−12 h	*2/day*	+	+	+	+	–	+	+	No pregnancy	CZP
II-10	<2	30 m−6 h	*1/day*	+	+	+	+	+	–	+	Improvement	CZP
II-17	<2	2 m−10 h	*1–2/day*	+	+	–	–	–	–	+	NA	CZP
II-18	<2	<2 h	Daily	+	+	+	+	–	–	+	Improvement	NA
III-6	* <2*	*10 m−1 d*	*2/day*	+	+	–	–	–	–	+	NA	CZP
III-8	* <2*	*10–15 m*	*Weekly*	+	+	–	–	–	–	–	No pregnancy	NA
III-12	* <2*	*NA*	*2/month*	+	+	–	+	–	–	+	NA	CZP
**Kwang-Dong Choi et al**.												
26	*53*	*Sec-Min*	*NA*	NA	+	NA	–	+	NA	NA	NA	NA
28	*49*	*Hours*	*NA*	NA	+	NA	+	–	NA	NA	NA	NA
**Gaudio A. et al**.												
II-1	* <1*	*Min-hours-day*	*Daily*	+	–	+	–	–	+	–	Marked improvement	PRG
III-1	* <1*	*Min-hours-day*	*Daily*	+	–	–	–	–	+	–	NA	CBZ

AAO, age at onset; 1/day, 1 per day; daily, multiple episodes per day, every day; NA, not available; CZP, clonazepam; PRG, pregabalin; CBZ, carbamazepine.

## 2. Case report

### 2.1. Patients

The proband was referred to our neuromuscular clinic for a hereditary myopathy. She was born from unrelated parents. Her mother is healthy, and her father reported with cramps, myalgia, and elevated serum CK levels since childhood. The father died when he was 60 years old due pneumonia complications. The proband reported frequent episodes of cramps in the lower limbs, especially at night, easy fatigability, and muscle weakness associated with increased serum CK levels (2–3 times the upper normal limit) since the age of three. The symptoms are triggered by mild physical exercise, such as a short walk, tiredness, and stress. A muscular biopsy was performed at the age of 12, which did not show any pathological finding. At the age of 22, none of the muscular CT scan, anti-Ach antibodies, or echocardiography showed pathological results. The EMG showed decreased motor unit action potential (MUAP) amplitude during maximal voluntary contraction, and the ischemia-hyperpnea test revealed double discharges (doublets) of motor units with spontaneous resolution. She was then diagnosed with spasmophilia and treated with pregabalin, with improvement of the neuromuscular symptoms. At the age of 25, during pregnancy, she had a strong improvement of symptoms, despite interrupting the therapy. She gave birth to a healthy male, later evaluated at a pediatric hospital at the age of one for myalgia and elevated serum CK levels (915 U/L; NV <150 U/L). The neurological examination at the age of 27 was unremarkable except for muscular hypertrophy, especially in the lower limbs. At the last examination (28y), the proband reported palpebral and hand muscle myokymia and frequent falls due to sudden muscular failure. The neurological examination was normal, except for diffuse muscle hypertrophy, especially in the lower limbs and mild difficulties in standing up from a squatting position. The muscular strength and reflexes were normal, and no rippling or myotonic phenomenon was observed. The gait was unremarkable, while run and jump were slightly impaired. Clonazepam was included in therapy, instead of pregabalin, with improvement of symptoms.

Since the age of 3, her son has daily episodes of inconsolable crying, mostly at night, due to legs cramps, easy fatigability compared to healthy peers, and sporadic falls. The EMG and Electrocardiogram (ECG) were normal, and the muscle biopsy showed no pathological findings.

### 2.2. Molecular analysis

Informed consent was obtained from proband and relatives for all genetic investigations, according to Regione Liguria ethical committee guidelines. Myotonic dystrophy type 2 (*ZNF9*), as well as duplications/deletions of the Duchenne Muscular Dystrophy (DMD) gene in both the proband and her father were screened according to an established protocol for hyperCkemia (9) and the results were negative. Myotonic dystrophy type 1 genetic testing was also negative. A two-step custom Next Generation Sequencing (NGS) gene panel analysis was performed on the proband and her son. The first panel included 52 genes associated with myotonia and periodic paralysis; the second included 67 genes associated with hyperCkemia and limb girdle muscular dystrophies. Both results were negative.

Array comparative genomic hybridization (aCGH) analysis was performed in the child and his parents using Agilent Human Genome G3 SurePrint 4 × 180 K Microarray (Agilent Technologies, California, USA) and analyzed by Genomic Workbench (Agilent), which revealed a paternal duplication of 0.4–0.5 Mbp on 15q25.2(chr15:83913016-84363679 × 3—GRCh37). The Copy Number Variant (CNV) classified as Variant of Unknown Significance (VUS) included *BNC1, SH3GL3*, and *ADAMTSL3*, genes not associated with neuromuscular disorders.

Since family history was indicative of an inherited disorder, a WES analysis was performed. Genomic DNA of the proband and her child was isolated from peripheral blood according to standard protocols. DNA was enriched and the exonic sequences were captured with SOPHiA Whole Exome Solution kit (SOPHiA Genetics^TM^) and sequenced on Illumina Novaseq6000 (Illumina^TM^). Sequencing data were cleaned, processed, and aligned against hg38 genome assembly. Quality Control (QC), alignment, variant calling, and annotation were performed using Sophia DDM v.4 software (SOPHiA Genetics^TM^). Variants were filtered and retained according to mean read depth > 10x, allele frequency in The Genome Aggregation Database (gnomAD) population <1%, allele frequency in an internal cohort <0.1%, segregation, and association with neuromuscular diseases. Eventually, only two variants were retained and prioritized according to American College of Medical Genetics and Genomics (ACMG) classification, co-segregation, and phenotype match. Both were validated via Sanger sequencing on an Abi Prism 3130XL sequencer (Thermofisher^TM^).

## 3. Results

**Figure 1:**
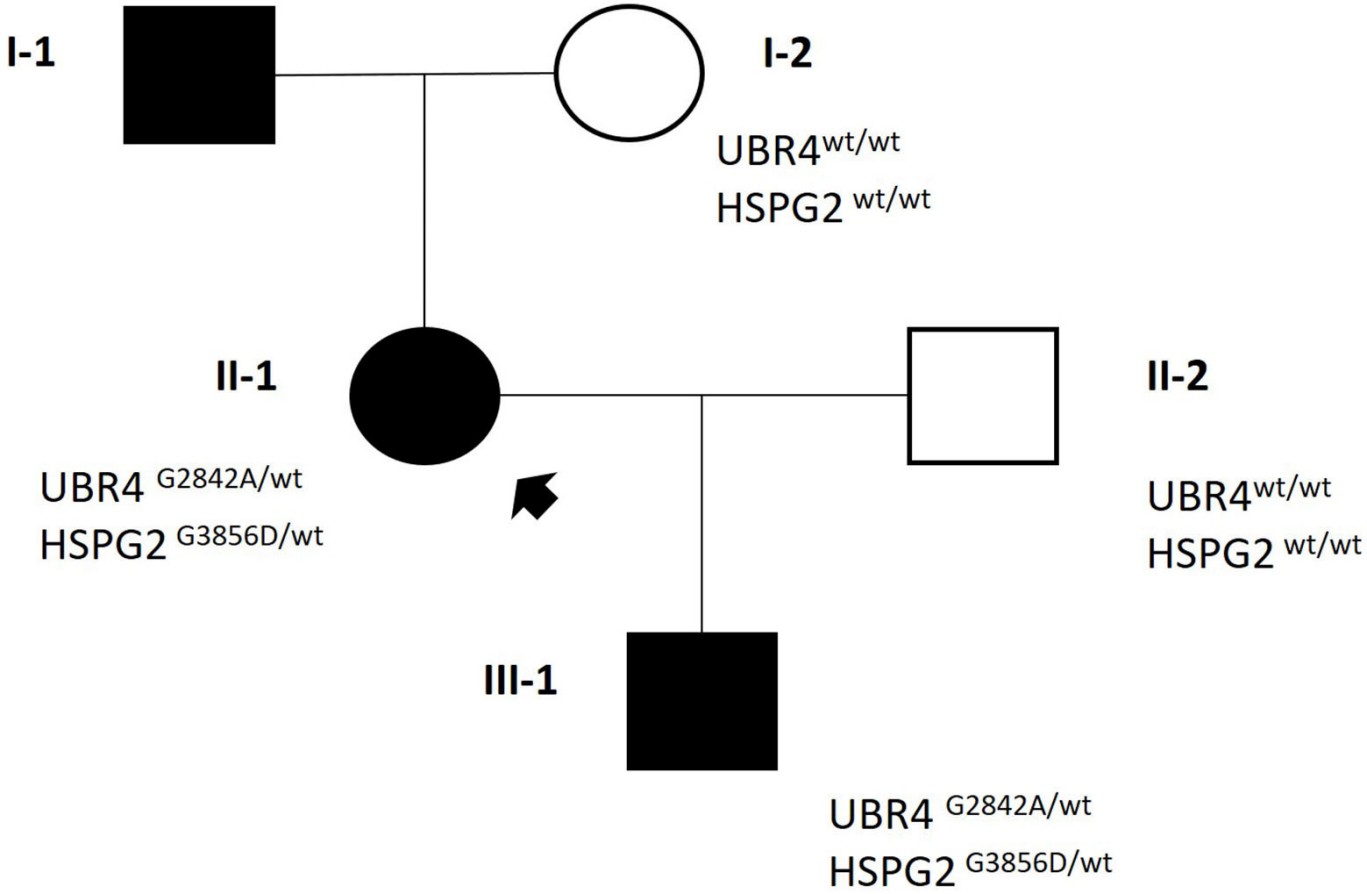
Family pedigree.

WES analysis revealed the co-segregation of two missense heterozygous variants in *UBR4* and *HSPG2*, namely, chr1:19140856:C>G—NM_020765.3: c.8525G>C (p.Gly2842Ala) and chr1:21831086:C>T—NM_005529.7: c.11567G>A (p.Gly3856Asp). Both variants have very rare frequencies in general population databases. Notably, computational *in-silico* tools unanimously predicted the pathogenicity of HSPG2 variant. Based on this evidence, both variants were classified as VUS, according to ACMG criteria (*UBR4*: PM2m, PP2s. *HSPG2*: PM2m, PP3s [Revel]). To evaluate family segregation, Sanger sequencing was performed on the proband's healthy mother, who resulted wild-type in both loci (Figure 1).

## 4. Discussion

Here, we present the case of a family who was referred to our clinic due to attacks of muscle cramps, pain, slight increased serum CK levels, hypertrophy, and transient muscle weakness. Although the cardinal features of myotonic syndromes (e.g., warm-up phenomenon and myotonic contraction) were missing, the set of symptoms and signs suggested a possible diagnosis of a genetically determined non-dystrophic myotonia-like disorder. However, the apparent myopathic origin of these clinical features were not confirmed by instrumental and morphological assessments (EMG, muscle CT scan, and muscle biopsy). Given the family history was suggestive of an inherited disorder, genetic testing was extensively performed, and WES analysis revealed the co-segregation of two variants in *UBR4* and *HSPG2*, genes previously identified in a novel subtype of EA (5). Although the family phenotype was not clearly suggestive of EA, the molecular findings, and some peculiar clinical overlap with EA8 (e.g., cramps, muscle weakness, improvement during pregnancy, and responsiveness to clonazepam), led to a careful review of the available literature of this group of disorders.

EAs are a group of clinical and genetic multifaceted disorders, occasionally lacking its core feature, i.e., ataxia (10). Sometimes, the wide phenotypic spectrum is related to the variant's functional effect (gain- or loss-of-function), e.g., *CACNA1A* or *SCNA2*; sometimes, phenotypes are highly variable, even within the same functional effect, e.g., EA1 patients carrying p.T226R substitution differ from p.T226A or p.T226M carriers (11). Moreover, intra-familiar phenotypic variability is also reported (12), even in monozygotic twins (13). The broad extension of the clinical spectrum is often documented in single case reports with an atypical phenotype (4), raising doubts over the actual association of the clinical features with the reported variant, especially in the absence of extensive genetic studies. Furthermore, genetic modifiers as well as environmental factors are likely to influence the clinical outcome, but both have been poorly investigated so far (11).

According to Online Mendelian Inheritance in Man (OMIM), heterozygous missense variants in *UBR4* are associated with EA8.

EA8 was first described in 2014 in a large Irish family with a rather different phenotype compared with the previously described EAs (4, 5, 11). The affected members manifested EA within the second year of life. The episodes were characterized by ataxia, dysarthria, nystagmus, myokymia, ocular twitching, and tremor and usually lasted minutes to hours, triggered by stress or tiredness. Peculiar features were the improvement of symptoms during pregnancy and the unusual responsiveness to clonazepam while being unresponsive to acetazolamide (4, 11). Linkage analysis identified a novel locus in 1p36.13-p34.3. Subsequent WES analysis and prioritization identified heterozygous missense variants in *UBR4* and *HSPG2*. *UBR4* was prioritized as the potential candidate since: (a) UBR4 had been reported in association with Ca2+-Calmodulin complex, as well as interacting with ITPR1, an Inositol 1,4,5-triphosphate receptor, both important in calcium signaling and thus potentially involved in the regulation of neuronal excitability (14); and (b) biallelic *HSPG2* variants are associated with two recessively inherited disorders (dyssegmental dysplasia, Silverman–Handmaker type #MIM 224410; Schwartz–Jampel syndrome, type 1 #MIM 255800) while heterozygous carriers are asymptomatic.

Recent studies on drosophila highlighted the importance of UBR4 in the protein synthesis/degradation pathway (15–18). Being an E3-Ubiquitin ligase, its dysregulation affects the degradation of misfolded or corrupted proteins via proteosome. Moreover, the same model demonstrated *UBR4* activity in promoting protein synthesis via direct impact on mRNA translation. The authors demonstrated that damaging *UBR4* variants impair the balance between protein synthesis and degradation, promoting muscle hypertrophy via either increased protein synthesis or decreased ubiquitination and recycle. This supports the importance of UBR4 in signaling and in axonal and muscle development and function. However, to date, no functional study has been performed to establish a clear and exclusive role of *UBR4* in EA8. Thus, the co-segregation of *UBR4* and *HSPG2* variants in the affected individuals cannot be ruled out and it may suggest that they play a synergistic role in EA8 pathogenesis.

Additionally, although rarer, milder phenotypes have been observed in Schwartz–Jampel syndrome in which the major signs are mostly myopathic (19). This strengthens the idea of a potential role of *HSPG2* in the variable clinical manifestation of EA8.

*HSPG2* encodes for perlecan, an extracellular matrix (ECM) proteoglycan (20, 21) fundamental for acetylcholinesterase (AchE) anchoring onto the neuromuscular junction (NMJ) (21–23). The complex perlecan's quaternary structure facilitates the binding of different extracellular molecules, such as integrins and α-destroglycan. When bound, perlecan becomes able to bind the AchE, anchoring it to the correct position at the NMJ. Studies revealed that an aberrant dosage of perlecan in the ECM affects its ability to correctly anchor the AchE to the NMJ (24), dysregulating AchE activity and preventing Ach firing and signaling (24). This has been suggested as the candidate pathomechanism underlying the two *HSPG2*-associated conditions with consequences on muscles such as cramps, easy fatigability, weakness, and progressive wasting (21, 24).

Since the initial report, another group identified disease-causing variants in *UBR4* in two unrelated Korean cases, but the co-segregation with other variants, such as *HSPG2*, has not been investigated (25). These two cases differed from the Irish patients in age at onset and, in one patient, ataxia was not the major sign. Furthermore, Kwang-Dong and colleagues identified two additional unrelated patients with a missense heterozygous variant in *UBR4* and a pathogenic mutation in *CACNA1A*. The clinical presentation was different from *CACNA1A*-associated EA2 patients and from the two with just a heterozygous missense variant in UBR4, especially in the earlier age at onset. The authors hypothesized a possible role for *UBR4* as a genetic modifier for *CACNA1A* (25).

However, no functional study has been performed either to clearly establish the pathogenic involvement of *UBR4* and *HSPG2* in this disorder, nor to rule it out. It is still unclear whether or not they are linked to such a phenotype, and whether they act alone or synergistically in the pathogenesis. Given the above-mentioned wide variability, we hypothesized that in these condition modifiers do exist and that, in the reported family, *HSPG2* could act as a possible genetic *UBR4* modifier. The two encoded proteins share common pathways at the NMJ level, namely, signaling transduction. It is possible that not-completely functional proteins exert synergistic damage at different cellular and tissue levels depending on the position and consequence of the mutation, thereby varying the clinical manifestation. Additionally, we cannot rule out environmental modifiers, which are, unfortunately, more difficult to identify due to the limited patient cohort availability.

To date, the description of the EA8 clinical spectrum is highly variable and, unfortunately, is limited to just two families. It typically manifests in infancy, but adulthood age at onset has been described as well. The major symptom is episodic ataxia, which typically manifests as recurrent episodes of unsteadiness, clumsiness, and coordination difficulties. Moreover, frequent cramps and extensive muscle weakness have been described. The duration and frequency of these attacks vary from seconds to minutes, sometimes they can last for days to months. There is no clear definition of how an attack primarily manifests and distinguishing symptoms during attacks can be challenging, especially when episodes occur in close temporal proximity. Furthermore, it is unclear what triggers such attacks. Some triggers have been identified as physical exercise, stress, and changes in temperature, but, as well as in the other EA subtypes, there could be many more, including food, immunological status, and others that are yet to be identified. Some peculiar features highlighted by Conroy et al. are the marked improvement of symptoms during pregnancy and the lack of responsiveness to acetazolamide, which represents the most effective drug in the other EAs subtypes.

The family described here present no episodes of ataxia. The main clinical characteristics are frequent muscle cramps, often after mild physical activity, muscle hypertrophy, and sudden muscle weakness that culminates in falls. It is unclear if this manifestation is continuous or is the results of multiple temporally close attacks, which unfortunately are indistinguishable. Triggers can be identified in physical exercise and stress, but the influence of other secondary factors cannot be ruled out. Notably, the proband referred a dramatic resolution of symptoms during pregnancy, even without any medical treatment in the meantime. Instrumental ascertainments excluded primary muscular damage. Muscle biopsy revealed no specific alteration. EMG and the ischemia-hyperpnea test highlighted a decreased amplitude of MUAP and doublets, resembling a neuromuscular failure of probable muscular genesis.

There are often no clear-cut edges between the clinical manifestations in neuromuscular disorders, and sometimes this complicates the definition of the correct phenotype, misleading both clinicians and geneticists on what to give attention to and investigate. The current paper aims to argue the precise and comprehensive clinical description, genetic determinants, and pathophysiology underlying this complex group, especially EA8, while also raising doubts over a possible clinical overlap between different conditions.

On the one hand, the genetic studies cannot exclude the presence of complex rearrangements and additional genetic defects in uninvestigated genomic regions; on the other, we cannot rule out the involvement of both *UBR4* and *HSPG2* in the definition of the clinical features. However, much more effort is needed in order to disentangle the precise underlying molecular mechanism.

The uncertainty in establishing a clinical diagnosis, coupled with incomplete knowledge of the molecular and genetic backgrounds in such phenotypes reflects in challenging genetic testing and counseling. Altogether, this complicates the exact clinical picture definition and the correct genotype–phenotype inference in these overlapping disorders. Episodic ataxias may be misdiagnosed for a variety of reasons, including phenotype–genotype variability, clinical overlap with primary and secondary causes, and common mimicking disorders. Therefore, it is crucial to extend the molecular study of these genes, even in undiagnosed patients with family recurrence of myopathic clinical features or with presenting phenotypes overlapping any features of this complex and heterogeneous group of disease. The more clear-cut the clinical diagnosis, the easier the identification of a possible genetic determinant, and the better the treatment and prognosis of the subject.

## Data availability statement

The datasets presented in this article are not readily available because of ethical and privacy restrictions. Requests to access the datasets should be directed to the corresponding author.

## Ethics statement

The studies involving humans were approved by Regione Liguria Ethical Committee. The studies were conducted in accordance with the local legislation and institutional requirements. Written informed consent for participation in this study was provided by the participants' legal guardians/next of kin. Written informed consent was obtained from the minor(s)' legal guardian/next of kin for the publication of any potentially identifiable images or data included in this article.

## Author contributions

AGa, FG, and PM designed and supervised the study. CP, FS, LT, CG, CC, GA, CF, and MG obtained consent from patients and performed the clinical evaluation and description. AGa, AGe, SP, and SG set up molecular and bioinformatics analysis. AGa, FG, CP, and PM wrote sections of the manuscript. All authors contributed to manuscript revision, read, and approved the final manuscript.
